# Real-world characteristics of women with endometriosis-related pain entering a multidisciplinary endometriosis program

**DOI:** 10.1186/s12905-020-01139-7

**Published:** 2021-01-07

**Authors:** Sanjay K. Agarwal, Oscar Antunez-Flores, Warren G. Foster, Ashwaq Hermes, Shahrokh Golshan, Ahmed M. Soliman, Amanda Arnold, Rebecca Luna

**Affiliations:** 1 Center for Endometriosis Research and Treatment, Department of Obstetrics, Gynecology and Reproductive Sciences, University of California San Diego, 9500 Gilman Drive, #0633, La Jolla, CA 92093-0633 USA; 2 Women’s Health, US Medical Affairs, AbbVie Inc., Chicago, IL USA; 3 Department of Obstetrics and Gynecology, and the School of Biomedical Engineering, McMaster University, Hamilton, ON Canada; 4 Department of Psychiatry, University of CA, San Diego, San Diego, CA USA; 5 Health Economics and Outcomes Research, AbbVie Inc., Chicago, IL USA

**Keywords:** Endometriosis, Pain, Real-word evidence, Multidisciplinary approach

## Abstract

**Background:**

Women with endometriosis are commonly treated by their sole provider. In this single-provider model of care, women frequently report long diagnostic delays, unresolved pelvic pain, multiple laparoscopic surgeries, sequential consultations with numerous providers, and an overall dissatisfaction with care. The emergence of multidisciplinary endometriosis centers aims to reduce diagnostic delays, improve pain management, and promote patient satisfaction; however, baseline data at the time of presentation to a multidisciplinary center are lacking.

**Methods:**

A real-world, retrospective, single-site, cross-sectional study of women with surgically confirmed and/or clinically diagnosed endometriosis generated baseline data for a planned longitudinal assessment of multidisciplinary care of endometriosis. The primary objective was to determine the proportion of patients experiencing mild, moderate, or severe pain for dysmenorrhea, non-menstrual pelvic pain (NMPP), and dyspareunia at entry into a multidisciplinary endometriosis clinic. Also explored were relationships between pain scores and clinical endpoints obtained from electronic medical records.

**Results:**

More than half (59%) of the study participants (*n* = 638) reported experiencing pelvic pain for ≥ 5 years. Pain intensity was highest for patients reporting dysmenorrhea, followed by NMPP, and dyspareunia. Significant correlations were observed between total pelvic pain and patient age (*r* = –0.22, *p* < 0.001, *n* = 506) and number of previous healthcare providers (*r* = 0.16, *p* = 0.006, *n* = 292); number of previous providers and duration of pain (*r* = 0.21, *p* = < 0.0001, *n* = 279); and duration of pain and years since diagnosis (*r* = 0.60, *p* < 0.001, *n* = 302). Mean pain scores differed significantly by age group for dysmenorrhea (*p* < 0.001), NMPP (*p* = 0.005), and total pelvic pain (*p* < 0.001), but not for dyspareunia (*p* = 0.06), with the highest mean pain scores reported among those < 30 years of age.

**Conclusion:**

These real-world data indicate that in the single-provider model of care, unresolved pelvic pain is common among women with endometriosis. Alternative care models, including a multidisciplinary approach, need to be evaluated for improvements in clinical outcomes. These data also highlight the importance of addressing NMPP, which may be particularly troublesome for patients.

## Background

Endometriosis is a chronic, inflammatory, and estrogen-dependent condition characterized by the implantation of endometrial-like tissue outside the uterus and is associated with pelvic pain and subfertility [1]. An estimated 5–10% of reproductive-age women, or approximately 176 million women worldwide, are affected by this disease [2–5]. Pelvic pain due to endometriosis is typically chronic and persists for more than 6 months. Although typical endometriosis pain symptoms include dysmenorrhea, non-menstrual pelvic pain (NMPP), and dyspareunia, others, including abdominal or back pain, dyschezia, and bloating, are also common and may lead to unnecessary testing and treatment, which could impede correct diagnosis [1, 3, 6].

Patients with chronic pelvic pain display an array of symptoms, potentially related to several common gynecologic and non-gynecologic conditions, making timely diagnosis of endometriosis challenging and resulting in diagnostic delays of up to 12 years [1, 7, 8]. Delays in diagnosis lead to delayed implementation of effective treatment, including treatment of pain and infertility associated with endometriosis, which negatively impact the patient’s quality of life and result in substantial economic burden stemming from higher healthcare utilization expenses [9]. Diagnostic delays may be due to multiple factors, including normalization of pelvic pain and dysmenorrhea, misinterpretation of symptoms as being due to other comorbidities such as depression, inflammatory bowel disease, interstitial cystitis, among others, as well as the need for laparoscopic diagnosis and frequent referrals to other specialists.

Another potential reason for the observed diagnostic delay may be that women with endometriosis conventionally seek and receive treatment from a single healthcare provider, often their usual gynecologist or primary care physician [10, 11]. This single-provider model of care may pose challenges in diagnosis and treatment for both the patient and provider for many reasons including mischaracterization of “normal” menstrual pain and the presence of non-specific symptoms. In addition, under the single-provider model, women with comorbidities, such as inflammatory bowel or bladder disease, nociceptive pain, and mental health disorders, are generally referred to providers who specialize in treating those conditions but lack expertise in diagnosing and treating endometriosis. Taken together, the result may be a delay in the diagnosis of endometriosis and suboptimal patient-focused comprehensive management. Other limitations of the single-provider model include inconsistent or delayed referral to other specialists, consultations with multiple providers for diagnosis, and lack of multimodal or holistic treatment [10, 12–14]. Effective treatment for endometriosis and any associated conditions may additionally be hindered by a lack of coordinated care by the patient’s various providers. As a result, an estimated 70% of women receiving care for endometriosis under the single-provider model report unresolved pain [6].

Conversely, a multidisciplinary model of care, leveraging the expertise of practitioners in multiple domains who are familiar with the treatment of endometriosis, has been proposed as an alternative treatment paradigm with the potential to improve outcomes for women with endometriosis [10, 13]. With this management approach, multiple care team specialists with expertise in specific therapeutic areas as well as broad expertise in the disease of interest (in this case, endometriosis) work in a coordinated, patient-focused way to treat patients with endometriosis and associated comorbidities. Such an approach has proven very effective in multiple therapeutic areas, including diabetes, nephrology, neurology, and oncology, and results in faster time to diagnosis, an optimized patient referral process, and faster receipt of efficacious treatment [15–20]. Despite the promise of more effective multidisciplinary care for endometriosis, definitive longitudinal data assessing the efficacy of the multidisciplinary approach are lacking. An initial step in evaluating the efficacy of multidisciplinary care is to characterize endometriosis-related pain in a real-world population of women served by the traditional single-provider model of care. In the present study, we characterize demographics, clinical characteristics, and pain severity associated with dysmenorrhea, NMPP, and dyspareunia in women with either surgically confirmed endometriosis or clinical symptoms consistent with endometriosis, who have been previously treated by single providers, solely or sequentially, before entering a multidisciplinary treatment program, the goal being to provide baseline data and generating hypotheses for future investigation. In addition, we explore factors that are associated with opioid use and frequency of surgery.

## Methods

### Study design and patients

This study was a retrospective, single-site, cross-sectional, descriptive study of patients seeking care at the University of California San Diego Center for Endometriosis Research and Treatment (CERT) clinic between 2011 and 2018. All patients had previously received care from one or more single providers for endometriosis prior to enrolling in CERT for multidisciplinary care management. Women aged 16–55 years with surgically confirmed endometriosis and/or clinically diagnosed endometriosis were included. A clinical diagnosis of endometriosis was made based on the presence of symptoms suggestive of endometriosis in the absence of other explanations for the pain including adenomyosis, fibroids, ovarian cysts, and musculoskeletal abnormalities [12]. Clinical symptoms suggestive of endometriosis were defined by International Classification of Disease, 9th or 10th edition codes for endometriosis and included dysmenorrhea, NMPP, and dyspareunia. We excluded women who were pregnant, breastfeeding, or seeking fertility treatment, as well as those with confirmed alternative causes for their pelvic pain. This study was approved by the Institutional Review Board at the University of California San Diego (approval number 181610).

### Data collection and assessments

Data from the initial CERT consultation were abstracted from electronic medical records (Epic, Verona, WI, USA) for all patients meeting inclusion/exclusion criteria and stored in a Microsoft Access (Microsoft Corporation, Redmond, WA, USA) database. Because the objective of this study was to gain insight into the patients’ experiences within the single-provider model of care, before entry into our multidisciplinary program, data abstraction was limited to the initial visit in CERT. Patient characteristics included age at entry into the CERT clinic, race, body mass index (BMI), family history of endometriosis, current smoking status, and history of alcohol consumption. Medical history variables included type of pain (dysmenorrhea, NMPP, and dyspareunia), pelvic or abdominal tenderness, length of time since onset of pain and diagnosis, duration of pain, and prior endometriosis-related surgeries at non-specialist centers, as well as current and prior use of medications for endometriosis-related pain. Duration of pain was defined as the length of time between onset of pain and entry into the CERT clinic. Prior use of medications was defined as those medications used for the management of symptoms since onset of symptoms and prior to the time of presentation. We also captured the number of previous healthcare providers consulted since the onset of symptoms for the management of pelvic pain before entry into the CERT clinic. Results from transvaginal ultrasound imaging for clinically significant comorbid conditions were abstracted and included ovarian cysts greater than 2 cm in size, fibroids greater than 3 cm in size, adenomyosis, and other potential causes of pelvic pain, such as hydrosalpinx. Endometriomas greater than 2 m in size were also noted. Operative reports were obtained for patient’s prior surgeries.

Pain severity was assessed using patient responses on the modified Biberoglu and Behrman pain scale questionnaire, which patients completed on their entry into the CERT clinic. The Biberoglu and Behrman scale was modified to capture only the three patient-reported pain symptoms (dysmenorrhea, NMPP, dyspareunia) [21]. Consistent with previous use of the Biberoglu and Behrman scale, patients reported maximum pain severity over the preceding month for dysmenorrhea, NMPP, and dyspareunia on a scale of 0 to 3, where higher numbers indicate more severe symptoms. To improve consistency and reduce inaccuracy in patient response, patients were read descriptions for the scores (0 to 3) of the pain scales for dysmenorrhea, NMPP, and dyspareunia. As an example, for dyspareunia, no pain scored 0, tolerated discomfort during sex scored 1; pain interrupted sex scored 2; and pain prevented sex scored 3. Patients were asked to choose the score corresponding to the text that best applied to them. Total pelvic pain was defined as the sum of scores for dysmenorrhea, NMPP, and dyspareunia, and thus ranged from 0 to 9. Medical and surgical histories, medication use, and pain severity assessments were recorded by the same physician (S.A.) at patients’ initial visits to the CERT clinic.

### Statistical analysis

Demographic and medical history variables were summarized using descriptive statistics. The mean and standard deviation (SD) were calculated for the continuous pain severity scores for dysmenorrhea, NMPP, dyspareunia, and total pelvic pain. Pearson’s correlation coefficients were computed to identify potential associations between total pelvic pain score and demographic and medical history variables. Chi-square tests, Pearson correlation, and analysis of variance were used to assess differences between groups in stratified analyses for categorical and continuous variables, respectively. Analysis of variance was also used to examine relationships between clinical variables and opioid use and frequent surgery. Analyses were conducted using IBM SPSS Statistics for Windows, Version 24 (IBM Corp [released 2016], Armonk, NY, USA) and results were considered statistically significant when *p* < 0.05.

## Results

### Patient characteristics and medical histories

Of the 1004 records reviewed, 366 were excluded for not meeting entry criteria or for having a predefined exclusion criterion. The resulting study population included for analyses comprised records from 638 women with either a surgically confirmed (*n* = 392) or clinical (*n* = 246) diagnosis of endometriosis. Patients mean age at entry into the CERT clinic was 33 years with 75% being younger than 40 years (Table 1). Most patients identified as white and the mean BMI was 25.7 kg/m^2^. A family history of endometriosis was reported by 10% of patients. Of the study participants, 10% reported current smoking and 51% reported that they consumed alcohol.

**Table 1:** Baseline demographics

Characteristic	Single-provider cohort entering the CERT clinic (*n* = 638)
Age, years	
* n*	637
Mean (SD)	33.41 (8.18)
Range	16–54
Age category, *n* (%)	
< 30 years	220 (35)
30–39 years	254 (40)
≥ 40 years	163 (26)
Race, *n* (%)	
White	559 (88)
Black or African American	22 (4)
American Indian or Alaskan Native	4 (1)
Asian	44 (7)
Native Hawaiian or Pacific Islander	3 (1)
BMI, kg/m^2^	
* n*	620
Mean (SD)	25.7 (6.2)
Range	15–62
BMI category, *n* (%)	
< 18 kg/m^2^	12 (2)
18 to < 25 kg/m^2^	352 (57)
26 to < 30 kg/m^2^	155 (25)
> 30 kg/m^2^	101 (16)
Family history of endometriosis, *n* (%)	
Yes	65 (10)
No	573 (90)
Smoking history, *n* (%)	
Yes	65 (10)
No	573 (90)
Alcohol consumption, *n* (%)	
Yes	327 (51)
No	311 (49)

At entry into the CERT clinic, most patients reported at least one of the classic symptoms consistent with endometriosis (dysmenorrhea, 80%; NMPP, 78%; dyspareunia, 58%), with 48% reporting all three symptoms (Table 2). As expected, commonly detected signs included pelvic and abdominal tenderness. Whereas hematuria and rectal bleeding were much less commonly observed. Relevant comorbidities identified from transvaginal ultrasound included uterine fibroids > 3 cm, adenomyosis, endometrioma > 2 cm, and other ovarian cysts > 2 cm in 10%, 7%, 17%, and 11% respectively. The mean time from onset of symptoms to diagnostic surgery was 6.3 ± 6.8 years, while the mean time from onset of symptoms to entry into the CERT clinic was 10.1 ± 9.0 years (range 0–40.1). The lower bound likely reflects incidental findings of endometriosis during surgery for other conditions. Patients reported an average of 6.9 ± 6.9 years between surgical diagnosis of endometriosis at a non-specialist center and their first CERT clinic visit. Half of the study population had been surgically diagnosed with endometriosis within 5 years and 59% had experienced pain for at least 5 years, prior to their initial consultation at CERT.

**Table 2:** Baseline disease characteristics and healthcare histories

Parameter	Single-provider cohort entering the CERT clinic (*n* = 638)
Type of pain, *n* (%)^a^	
Dysmenorrhea	511 (80)
Dyspareunia	369 (58)
NMPP	498 (78)
Additional symptoms, *n* (%)	
Pelvic tenderness	275 (43)
Abdominal tenderness	140 (22)
Hematuria	12 (2)
Rectal bleeding	38 (6)
Comorbidity identified by transvaginal ultrasound	
Uterine fibroids (> 3 cm)	
No	572 (90)
Yes	66 (10)
Suspected adenomyosis	
No	593 (93)
Yes	45 (7)
Suspected endometrioma (> 2 cm)	
No	530 (83)
Yes	108 (17)
Other ovarian cysts (> 2 cm)	
No	565 (89)
Yes	73 (11)
Years since onset of pelvic pain to endometriosis surgical diagnosis	
* n*	285
Mean (SD)	6.3 (6.8)
Range	0–32
Years from surgical diagnosis of endometriosis to entry into the CERT clinic	
* n*	392
Mean (SD)	6.9 (6.9)
Range	0.02–35.2
Years from onset of pelvic pain to entry into the CERT clinic	
* n*	527
Mean (SD)	10.1 (9.0)
Range	0–40.1
Duration of pain, *n* (%)	
< 5 years	215 (41)
5–10 years	83 (16)
> 10 years	229 (44)
Years since diagnosis, *n* (%)	
< 1 year	77 (20)
1–5 years	122 (31)
5–10 years	90 (23)
> 10 years	103 (27)
Prior surgical procedures to treat endometriosis^b^, *n* (%)	670 (100)
Hysterectomy	42 (6)
Left oophorectomy	35 (5)
Right oophorectomy	16 (2)
Bilateral oophorectomy	5 (1)
Unilateral cystectomy	76 (11)
Bilateral cystectomy	16 (2)
Conservative surgery	480 (72)
Analgesic use, *n* (%)	
NSAIDs	
Current	219 (34)
Prior	86 (14)
Opioids	
Current	158 (25)
Prior	108 (17)
Combination NSAID/opioid^c^	
Current	84 (13)
Endometriosis medication use, *n* (%)	
Oral contraceptives	
Current	177 (28)
Prior	391 (63)^d^
GnRH agonist (Lupron)	
Current	26 (4)
Prior	120 (19)
Depo provera	
Current	17 (3)
Prior	80 (13)
Oral progestin	
Current	36 (6)
Prior	40 (6)
Danazol	
Current	1 (0.2)
Prior	4 (1)
IUD	
Current	20 (3)
Prior	54 (9)
Aromatase inhibitors	
Current	5 (1)
Prior	7 (1)
Gabapentin	
Current	18 (3)
Prior	8 (1)
Antidepressant	
Current	125 (20)
Prior	39 (6)
Previous healthcare providers, *n*	638
Mean (SD)	4.0 (3.7)
Range	0–26

Percentages reflect the number of patients with non-missing data. Numbers may not add up to 638 because of missing data

^a^The number (%) of patients reporting mild, moderate, or severe pain for each condition. Total number as follows: dysmenorrhea, *n* = 569; NMPP, *n* = 571; dyspareunia, *n* = 551

^b^Data based on the five most recent surgeries. Women described here may have had more than one surgery and so more than one procedure

^c^Prior use of NSAIDS and opioids at the same time could not be confirmed accurately

^d^Prior oral contraceptive use includes a very few women who used etonogestrel/ethinyl estradiol vaginal ring

Of the 386 women who had been surgically treated and provided the number of endometriosis surgeries they had undergone since their initial diagnostic surgery, 226 (59%) reported one surgery and the remaining 160 (41%) reported repeat surgeries with 93% reporting three or fewer surgeries. While most patients had not undergone repeat surgeries to treat endometriosis, those treated surgically underwent an average of 1.7 ± 1.4 endometriosis surgeries (range 1–18) from diagnosis to entry into the CERT clinic. Interestingly, left oophorectomy was more common (5%) than right oophorectomy (2%) in this patient population. Of those treated surgically, the mean number of endometriosis surgeries per year since diagnostic surgery was 0.5 ± 0.4, (range 0.030–52.1). The patient reporting 52.1 surgeries per patient-year visited CERT 1 week following her surgery, and thus represents an outlier, highlighting a limitation of this type of analysis.

Patients received healthcare for endometriosis from an average of four previous healthcare providers (range 0–26) prior to entry to the CERT clinic, regardless of when they were diagnosed with endometriosis. The most common previous healthcare providers reported by patients entering CERT were obstetrician/gynecologists (512 [80%]) or primary care physicians (276 [43%]). Emergency room physicians and gastroenterologists were seen by 93 (15%) and 63 (10%) patients, respectively. Approximately one-third of the study population reported current analgesic use (non-steroidal anti-inflammatory drugs [NSAIDs], opioids) (Table 2). Approximately one-quarter of the study participants reported current use of opioids, whereas prior use of opioids was slightly lower (17%). At 34%, current ongoing use of NSAIDs was more frequently reported than opioids, although prior regular NSAID use (14%) was less common. Current use of combination NSAIDs/opioids was reported by 13% of study participants. Current oral contraceptive and antidepressant use was reported by roughly one-quarter (28% and 20%, respectively) of patients. Current use of other medications commonly used in the management of endometriosis was reported infrequently. These medications are shown in Table 2.

### Pain assessment

**Fig. 1:**
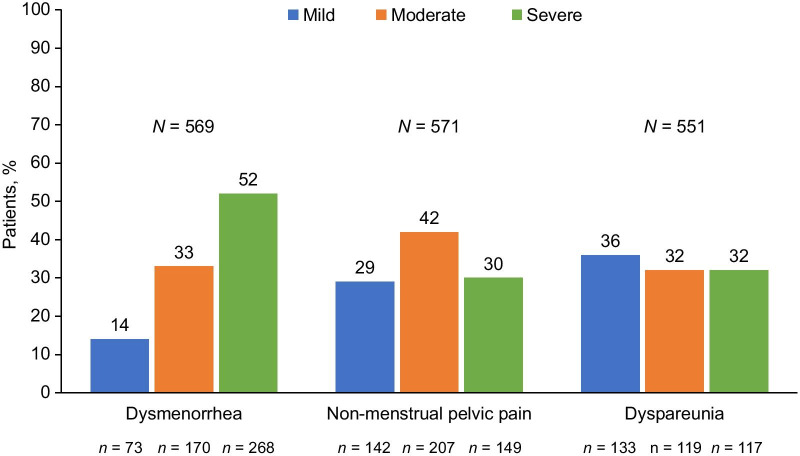
Proportion of patients stratified by pain severity score for dysmenorrhea, dyspareunia, and non-menstrual pelvic pain. The percentage of patients reporting mild (pain score = 1), moderate (pain score = 2), or severe (pain score = 3) pain are shown for each pain type. The number of patients reporting mild, moderate, or severe pain is shown below the bars. The total number of patients with data for each pain type (*N* shown above the bars) includes patients reporting no pain; percentages exclude patients in the “none” category

**Fig. 2:**
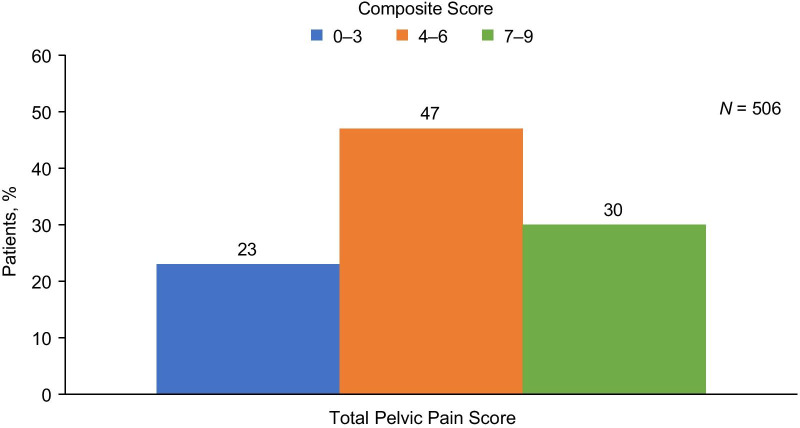
Proportion of patients stratified by categories of composite score for total pelvic pain. The percentage of patients reporting mild (composite score 0–3), moderate (composite score 4–6), or severe (composite score 7–9) pain is shown

Approximately half (52%) of the patients with dysmenorrhea reported severe pain, and most patients with NMPP reported moderate (42%) or severe (30%) pain, regardless of current treatment at the time of the initial CERT visit (Fig. 1). However, mild, moderate, and severe dyspareunia was reported in roughly equal frequencies. When scores for dysmenorrhea, NMPP, and dyspareunia were combined into the total pelvic pain score, most (77%) patients were categorized as experiencing moderate or severe pain (Fig. 2).

The mean (SD) total pelvic pain score on a 0–9 scale for the overall population was 5.22 ± 2.16. Patients reported higher pain scores for dysmenorrhea than they did for NMPP or dyspareunia (Table 3). When evaluating correlations between total pelvic pain score and clinical variables, it was detected that higher total pelvic pain was correlated with younger patient age (*r* = –0.22, *p* < 0.001, *n* = 506) and increased number of previous healthcare providers (*r* = 0.16, *p* = 0.006, *n* = 292). Significant correlations were also observed between the number of previous providers and time since onset of pain (*r* = 0.21, *p* = < 0.0001, *n* = 279) as well as time since onset of pain and years since surgical diagnosis (*r* = 0.60, *p* < 0.001, *n* = 302), confirming the assumption that patients with longer duration of pain saw more previous healthcare providers. Significant correlations with higher number of previous providers was due mainly to correlations with NMPP (*r* = 0.19, *p* = 0.001) rather than dyspareunia (*r* = 0.03, *p* = 0.58) or dysmenorrhea (*r* = 0.11, *p* = 0.06).

**Table 3:** Biberoglu and Behrman pain assessment stratified by select patient characteristics

Parameter, mean (SD)		Dysmenorrhea		NMPP		Dyspareunia		Total pelvic pain score (*n* = 506)
Overall		2.14 (1.00)		1.76 (0.98)		1.31 (1.14)		5.22 (2.16)
Age	*n*		*n*		*n*		*n*	
< 30	200	2.32 (0.91)	193	1.94 (0.88)	183	1.44 (1.05)	172	5.71 (1.86)
30–39	234	2.15 (0.95)	234	1.67 (0.99)	228	1.31 (1.13)	210	5.16 (2.16)
≥ 40	135	1.84 (1.13)	144	1.65 (1.06)	140	1.14 (1.25)	124	4.62 (2.38)
*p* value		< *0.001*		*0.005*		*0.06*		< *0.001*
*F* value		*9.53*		*5.35*		*2.89*		*9.57*
*df*		*2, 566*		*2, 568*		*2, 548*		*2, 503*
Number of previous providers	*n*		*n*		*n*		*n*	
0–1	60	1.93 (0.86)	61	1.62 (0.95)	59	1.07 (1.07)	57	4.58 (2.00)
2	57	2.25 (0.91)	58	1.66 (0.91)	5758	1.11 (1.08)	54	4.94 (1.93)
3	57	2.09 (0.98)	60	1.83 (0.91)	77	1.41 (1.18)	55	5.38 (2.08)
4–5	77	1.97 (1.11)	83	1.72 (0.99)	56	1.42 (1.16)	72	5.14 (2.31)
≥ 6	59	2.20 (1.11)	61	2.11 (0.90)		1.43 (1.16)	54	5.74 (1.95)
*p* value		*0.34*		*0.03*^a^		*0.18*		*0.04*^*a*^
*F* value		*1.13*		*2.76*		*1.56*		*2.48*
*df*		*4, 305*		*4, 318*		*4, 302*		*4, 287*
Duration of pain	*n*		*n*		*n*		*n*	
< 5	193	2.03 (1.00)	190	1.69 (1.00)	182	1.26 (1.12)	166	4.98 (2.21)
5–10	75	2.40 (0.87)	74	1.88 (0.94)	67	1.45 (1.18)	63	5.67 (1.82)
> 10	206	2.23 (0.95)	211	1.79 (0.96)	205	1.37 (1.14)	191	5.37 (2.04)
*p* value		*0.01*		*0.35*		*0.44*		*0.05*
*F* value		*4.57*		*1.06*		*0.82*		*2.97*
*df*		*2, 471*		*2, 472*		*2, 451*		*2, 417*
Years since diagnosis	*n*		*n*		*n*		*n*	
< 1	69	2.14 (1.06)	65	1.75 (1.00)	61	1.18 (1.16)	57	5.00 (2.29)
1–5	108	2.20 (1.00)	110	1.92 (0.95)	105	1.48 (1.16)	94	5.76 (2.11)
5–10	76	2.00 (1.14)	81	1.96 (1.03)	74	1.35 (1.17)	68	5.38 (2.34)
> 10	88	2.03 (1.08)	95	1.76 (0.95)	95	1.41 (1.22)	83	5.17 (2.26)
*p* value		*0.54*		*0.38*		*0.47*		*0.17*
*F* value		*0.72*		*1.02*		*0.85*		*1.67*
*df*		*3, 337*		*3, 347*		*3, 331*		*3, 298*
Current medications	*n*		*n*		*n*		*n*	
Opioids								
No	425	2.01 (1.00)	423	1.56 (0.93)	410	1.23 (1.13)	378	4.80 (2.13)
Yes	144	2.51 (0.86)	148	2.32 (0.90)	141	1.54 (1.14)	128	6.43 (1.74)
*p* value		< *0.001*		< *0.001*		*0.006*		< *0.001*
*F* value		*28.75*		*73.57*		*7.70*		*60.55*
*df*		*1, 567*		*1, 569*		*1, 549*		*1, 504*
NSAIDS								
No	373	2.05 (1.05)	379	1.68 (0.98)	358	1.28 (1.12)	335	5.03 (2.18)
Yes	196	2.31 (0.86)	192	1.91 (0.96)	193	1.36 (1.17)	171	5.58 (2.09)
* p* value		*0.004*		< *0.001*		*0.48*		*0.006*
* F* value		*8.57*		*6.80*		*0.51*		*7.66*
* df*		*1, 567*		*1, 569*		*1, 559*		*1, 504*
Ultrasound findings	*n*		*n*		*n*		*n*	
Fibroids								
No	511	2.16 (0.98)	517	1.77 (0.96)	497	1.33 (1.14)	459	5.27 (2.12)
Yes	58	1.95 (1.10)	54	1.67 (1.15)	54	1.13 (1.12)	47	4.70 (2.51)
* p* value		*0.12*		*0.48*		*0.22*		*0.09*
* F* value		*2.38*		*0.50*		*1.50*		*2.94*
* df*		*1, 567*		*1, 569*		*1, 549*		*1, 504*
Ovarian cysts								
No	506	2.13 (1.01)	503	1.78 (0.98)	490	1.32 (1.15)	448	5.24 (2.19)
Yes	63	2.19 (8.77)	68	1.59 (1.01)	61	1.21 (1.04)	58	5.00 (1.96)
* p* value		*0.66*		*0.13*		*0.48*		*0.42*
* F* value		*0.19*		*2.28*		*0.50*		*0.65*
* df*		*1, 567*		*1, 569*		*1, 549*		*1, 504*
Endometrioma								
No	470	2.14 (1.00)	475	1.81 (0.97)	454	1.34 (1.15)	417	5.31 (2.13)
Yes	99	2.11 (0.95)	96	1.47 (0.97)	97	1.16 (1.10)	89	4.75 (2.33)
* p* value		*0.76*		*0.002*		*0.17*		*0.03*
* F* value		*0.09*		*10.08*		*1.91*		*4.99*
* df*		*1, 567*		*1, 569*		*1, 549*		*1, 504*
Adenomyosis								
No	528	2.14 (1.00)	532	1.76 (0.98)	511	1.30 (1.14)	468	5.21 (2.15)
Yes	41	2.17 (1.00)	39	1.77 (1.01)	40	1.43 (1.15)	38	5.34 (2.27)
* p* value		*0.83*		*0.93*		*0.51*		*0.71*
* F* value		*0.05*		*0.01*		*0.44*		*0.14*
* df*		*1, 567*		*1, 569*		*1, 549*		*1, 504*

Pain assessment scores for dysmenorrhea, dyspareunia, and NMPP range from 0–3. Total pelvic pain score ranges from 0–9. Analyses include patients reporting no pain, mild pain, moderate pain, or severe pain for each condition. *p* values were obtained from the overall analysis of variance

^a^Overall analysis of variance was not significant. Significant differences between the 0–1 and six or more categories were demonstrated by appropriate post hoc comparison tests

To further investigate these correlations, we stratified the pain scores for dysmenorrhea, dyspareunia, NMPP, and total pelvic pain by patient age, number of previous providers, duration of pain, and years since diagnosis. We also stratified pain scores by analgesic use and ultrasound-identified comorbidities. Lower mean pain scores were reported by patients in older age categories for all pain types and total pelvic pain, with statistically significant differences between age categories observed for dysmenorrhea (*p* < 0.001), NMPP (*p* = 0.005), and total pelvic pain (*p* < 0.001) (Table 3). Patients with low total composite pain scores of 0–3 saw a mean of 3.1 ± 1.9 providers, while patients with medium and high total composite pain scores of 4–6 and 7–9 saw 4.0 ± 3.8 and 4.9 ± 4.8 providers, respectively. No overall significant differences by number of previous providers were observed for any of the pain types or total pelvic pain. However, in post hoc comparison, significant differences in pain score for NMPP (*p* = 0.03) and total pelvic pain (*p* = 0.04) were observed for patients who have previously seen ≥ 6 providers compared with those who have seen 0–1 provider. Significant differences by duration of pain were observed only for dysmenorrhea (*p* = 0.01), with the highest mean pain scores observed among those experiencing pain for 5–10 years. There were no significant differences in mean pain scores by years since diagnosis. Pain scores for dysmenorrhea, NMPP, and total pelvic pain differed significantly by opioid (*p* < 0.01) and NSAID (*p* < 0.01) use, while significant differences in pain scores for dyspareunia were observed only for opioid use (*p* < 0.01). When stratified by ultrasound-identified comorbidities of fibroids, adenomyosis, ovarian cysts, and endometriomas, significant differences in pain scores were observed only for endometrioma among patients with NMPP (*p* = 0.002).

### Relationships between clinical variables, opioid use, and surgery

#### Opioid use

Opioid use is an important issue with substantial societal and healthcare ramifications. The use of opioids was significantly associated with total pelvic pain score (*p* < 0.001), dysmenorrhea (*p* < 0.001), dyspareunia (*p* = 0.006), NMPP (*p* < 0.001), number of previous healthcare providers (*p* < 0.001), and number of previous surgeries before attending the CERT clinic (*p* < 0.02) (Table 4). Pain scores, number of previous providers, and number of previous surgeries were all significantly higher among current opioid users than non-users.

**Table 4:** Relationships between clinical variables, opioid use, and surgery

Parameter, mean (SD)	Opioid use				Surgery			
	Yes (*n* = 158)	No (*n* = 480)	*F* value	*p* value	Yes (*n* = 392)	No (*n* = 245)	*F* value	*p* value
Age, years	32.77 (8.10)	33.62 (8.20)	1.27	*0.26*	34.15 (8.03)	32.22 (8.28)	8.42	*0.004*
Weight, lbs	156.72 (44.2)	151.21 (36.5)	2.38	*0.12*	156.7 (41.6)	146.15 (32.28)	11.23	*0.001*
Height, in	64.36 (2.73)	64.80 (2.86)	2.89	*0.09*	64.71 (2.80)	64.66 (2.89)	0.05	*0.82*
BMI	26.47 (6.88)	25.37 (5.93)	3.71	*0.06*	26.31 (6.68)	24.61 (5.17)	11.26	*0.001*
Years from diagnostic surgery to entry into CERT	6.68 (6.55)	7.03 (7.00)	0.20	*0.65*	6.95 (6.90)	NA	NA	*NA*
Years from onset of pain to entry into CERT	10.94 (9.29)	9.86 (8.93)	1.41	*0.24*	11.35 (9.18)	8.49 (8.58)	13.17	< *0.001*
Number of prior surgeries	2.00 (2.06)	1.63 (1.03)	5.53	*0.02*	1.74 (1.40)	NA	NA	NA
Current opioid use	NA	NA	NA	NA	107 (27.3)	51 (2.70)	3.49	0.062
Dysmenorrhea score	2.51 (0.86)	2.01 (1.01)	28.75	< *0.001*	2.11 (1.05)	2.17 (0.91)	0.50	*0.481*
Dyspareunia score	1.54 (1.14)	1.23 (1.13)	7.70	*0.006*	1.39 (1.18)	1.18 (1.06)	4.63	*0.032*
NMPP score	2.32 (0.90)	1.56 (0.93)	73.57	< *0.001*	1.87 (0.97)	1.57 (0.97)	12.64	< *0.001*
Total pelvic pain score	6.43 (1.74)	4.80 (2.13)	60.55	< *0.001*	5.41 (2.23)	4.93 (2.02)	6.00	*0.015*
Number of prior healthcare providers	5.38 (5.14)	3.54 (2.94)	16.07	< *0.001*	4.31 (3.97)	3.44 (3.06)	4.36	*0.037*

*p* values were obtained from the overall analysis of variance

#### Surgery

Patients treated surgically were older (*p* = 0.004), had higher BMI (*p* = 0.001), and had been previously treated by a higher number of healthcare providers compared with those treated non-surgically. Mean pain scores for NMPP (*p* < 0.001), dyspareunia (*p* = 0.032), and total pelvic pain (*p* = 0.015) were higher among patients treated surgically, compared with those not treated surgically. No significant differences in mean pain score were observed for dysmenorrhea by surgical treatment status.

## Discussion

In this cross-sectional analysis, we examined the real-world demographics, clinical characteristics, and pelvic pain symptoms in women treated within the single-provider model of care. In addition to providing insights into current endometriosis care, these data will be used to establish baseline data for comparing outcomes in these women as they are treated within a multidisciplinary treatment program. This dataset is unique in that, although it is from a real-world patient population and clinical practice, the breadth of data captured is substantial and typically unavailable in real-world observational datasets. Our data from this retrospective study indicate that severe pain is a common experience among women with dysmenorrhea, NMPP, and dyspareunia, and that pain severity may be significantly greater among younger women. An average of 10 years between onset of pelvic pain and entry into the CERT clinic was observed, regardless of previous diagnosis and intervention.

Importantly, our data indicate that although dysmenorrhea was the most intense pain symptom experienced, it was NMPP that was the pain symptom most strongly associated with increased number of prior physicians consulted. The suggestion is that NMPP may be the most troubling pain symptom for patients with endometriosis and may be the one predominantly driving the patient-initiated search and referrals from primary physicians to other providers for a diagnosis and relief of pain. It is possible that this disparity between dysmenorrhea and NMPP can be explained by the fact that although dysmenorrhea is the most severe pain symptom, it is generally predictable in timing and so to some extent can be anticipated. Conversely, although NMPP may be less intense, it can be unpredictable and harder to manage, thus resulting in a greater problem for patients.

Our findings regarding pain severity in women with dysmenorrhea, NMPP, and dyspareunia are broadly in line with results published in previous studies [22–24]. In a study of 90 women undergoing laparoscopy for pelvic pain, severe pain was reported by patients with dysmenorrhea (24%), chronic pelvic pain (21%), and dyspareunia (3%) [22]. Data from the ENDO (Endometriosis, Natural History, Diagnosis, and Outcomes) study, in which patients recruited from 14 surgical centers in two states rated pelvic pain intensity on an 11-point visual analog scale, indicated that pain intensity for dyspareunia and dysmenorrhea varied by specific symptom, with mean dyspareunia scores ranging from 2.9 ± 3.3 (deep pain with intercourse) to 0.5 ± 1.6 (constant burning vaginal pain). Mean pain scores for dysmenorrhea ranged from 6.5 ± 3.2 (level of cramps with period) to 1.9 ± 2.9 (pain after period is over) [23]. In a study of 656 women receiving care at a tertiary referral center specializing in the interdisciplinary management of chronic pelvic pain and endometriosis, the mean (SD) chronic pelvic pain severity, rated on an 11-point scale, was 5.8 (3.2) [24]. Although our assessment rated total pelvic pain on a 0–9 scale and included pain associated with dysmenorrhea, NMPP, and dyspareunia, the mean total severity score we observed (5.22) was similar in magnitude.

The relationship between clinical variables, including pain scores, opioid use, and number of surgeries for endometriosis in our study population was examined. Within the healthcare community, and consistent with current guidelines from the American Society for Reproductive Medicine [25] and the American College of Obstetricians and Gynecologists [26], there is a general desire to reduce the frequency of invasive procedures performed for endometriosis. Current Centers for Disease Control guidelines also recommend reducing the use of highly addictive opioid medications for chronic pain management [27]. Indeed, it has been suggested that healthcare models of endometriosis care can be judged by clinical endpoints such as long-term pain and quality-of-life improvements over a minimum of 2 years, together with decreased emergency room visits and opioid use [10]. Reducing invasive procedures would also be consistent with those goals. In this analysis, we found that total pain scores, individual pain types, the number of previous surgeries, and the number of previous healthcare providers were all associated with opioid use, possibly suggesting inadequate pain management among patients with severe pain who have sought care sequentially from multiple providers. Results from recent studies have shown that women with endometriosis are at a nearly three-fold increased risk of using opioids for pain management compared with women without endometriosis [28], and that opioid use among women with endometriosis is associated with increased economic burden [29]. Given the substantial harms associated with opioid use, implementing a model of multidisciplinary care that incorporates complementary yet effective patient and symptom-centered pain management strategies (e.g., mental health programs; mind–body programs; optimizing the duration, retention, and discontinuation of treatments) may be critical for this vulnerable patient population.

In view of the chronic nature of endometriosis, the sub-optimal management of pelvic pain, and the number of endometriosis surgeries in single-care provider models, our findings highlight the need for a multidisciplinary care model that utilizes a patient-focused team of practitioners who have a depth of endometriosis knowledge plus a range of skills and expertise in a variety of disciplines. The goals of the multidisciplinary care model are to provide long-term, comprehensive, and individualized care in a coordinated and systematic fashion that lead to more effective management of this chronic condition. Within the CERT clinic, patients with endometriosis are treated by a team of physicians, surgeons, and other health professionals with expertise in reproductive endocrinology, infertility, gynecology, pain management, gastroenterology, urogynecology, general surgery, and mental health. Given that some pelvic pain may not be originating from endometriotic lesions but as a consequence of the disease itself (ie, neuropathic pain, pelvic floor dysfunction pain, vulvodynia, etc.), a coordinated multidisciplinary endometriosis center can also manage these other types of pain by providing integrative services including physiotherapy, acupuncture, and nutrition. Furthermore, patients benefit from staff members readily available to coordinate care between physician team members, which expedites timely referrals and ensures communication of clinical findings and patient management between the leading physician and other team members. Although endometriosis care at a large, experienced center is preferred, some specialist endometriosis care may require the establishment of satellite endometriosis clinics where additional interdisciplinary or multidisciplinary care services can be offered to ensure optimal whole-patient care for patients who reside further away from a large center.

Use of a multidisciplinary treatment paradigm has been evaluated for deep dyspareunia and chronic pelvic pain. Using an 11-point scale, Yong et al. evaluated the severity of deep dyspareunia among 278 women (84.9% with or suspected to have endometriosis) treated at a multidisciplinary center [30]. They found that after 1 year, the severity of deep dyspareunia had improved, with a reduction in the severe category (55.0% at baseline to 30.4% at follow-up) and increase in the absent-mild category (27.3% at baseline to 44.6% at follow-up). Allaire et al. demonstrated improvements in functional quality of life and a median two-point reduction in the severity of chronic pelvic pain from baseline to follow-up in a prospective study of 296 women treated for 1 year at a multidisciplinary center [31]. These data indicate that the multidisciplinary approach may improve outcomes in women with symptoms of endometriosis. However, a limitation of these studies is that women with and without endometriosis were included in the study population, as both deep dyspareunia and chronic pelvic pain can arise from multiple conditions, and the results may not be fully generalizable to the endometriosis clinical setting.

Women with endometriosis who are treated under the single-provider model of care face substantial hurdles in receiving effective treatment, including long diagnostic delays, unresolved pain, multiple physician consultations in search of diagnosis, lack of timely referrals to specialists, and lack of multimodal and/or holistic therapies. Multidisciplinary care is hypothesized to improve patient care and outcomes by removing some of these obstacles. Our analysis provides baseline data from a real-world clinical population of women with endometriosis previously treated by one or more single healthcare providers, which may be used in future studies to assess the efficacy of the multidisciplinary care approach in improving treatment outcomes for patients with endometriosis.

Limitations of our study include the cross-sectional, retrospective, and descriptive nature of the analysis. Because our data were captured at a single point in time, we could not evaluate changes in pain severity with disease progression or adjustments to pain medications. Patients were asked to report their medical histories and pain symptoms, which could result in misclassification due to inaccurate recall. The real-world data used for this analysis consistently lacked information on the use of magnetic resonance imaging to diagnose adenomyosis, thus, some of the reports of dysmenorrhea may have been due to adenomyosis. As data collection was limited to clinically relevant variables necessary for patient management, there may be other important variables affecting pain severity that we did not measure. Finally, our data were drawn from a population of women who were either self- or physician-referred to a single specialized center for endometriosis treatment and who may have had more severe symptoms than the overall population of women with endometriosis. Our findings may therefore not be generalizable to all other clinical settings.

Strengths of this study include the detailed assessment of pain and other clinical characteristics in a large, real-world clinical population. Importantly, detailed and consistently applied pain severity scores based on the modified Biberoglu and Behrman scale were available. Medical histories and pain assessment questionnaires were also collected with the same physician at entry into the CERT clinic, reducing the potential for misclassification due to interrater variability.

## Conclusions

Our current understanding of the burden of endometriosis and related symptoms is limited by the self-selected nature of the study populations in the published literature. Because the burden of disease may differ between women who do or do not choose to participate in research studies, such real-world data could improve our understanding of current practice patterns in the management of endometriosis and related pain. This study provides real-world baseline medical history and pain severity data for women entering our multidisciplinary endometriosis treatment program. The data suggest that the traditional, single-provider model of care may be insufficient for a substantial proportion of women who have endometriosis and that NMPP may be a particular problem for these patients, leading them to seek multiple opinions in search of satisfactory care. Given the consistent and increasing data illustrating the limitations of single-provider models of endometriosis care, alternative healthcare delivery models need to be sought. Multidisciplinary care has potential benefits. However, additional studies to prospectively examine clinically relevant treatment outcomes, such as rate of opioid use and persistent improvements in pain and quality of life, are needed to evaluate the efficacy of the multidisciplinary endometriosis care model.

## Data Availability

The datasets generated and/or analyzed during the current study are not publicly available due to patient privacy considerations and institutional IRB approval.

## Abbreviations

BMI: Body mass index
CERT: University of California San Diego Center for Endometriosis Research and Treatment
DF: Degrees of freedom
GI: Gastrointestinal
IUD: Intrauterine device
GnRH: Gonadotropin-releasing hormone
NA: Not applicable
NMPP: Non-menstrual pelvic pain
NSAID: Non-steroidal anti-inflammatory drug
SD: Standard deviation
